# Global sensitivity analysis of parameters based on sPCE: The case study of a concrete face rockfill dam in northwest China

**DOI:** 10.1371/journal.pone.0290665

**Published:** 2023-08-31

**Authors:** Li Ran, Jie Yang, Chunhui Ma, Lin Cheng, Mingjuan Zhou

**Affiliations:** 1 State Key Laboratory of Eco-hydraulics in Northwest Arid Region, Xi’an University of Technology, Xi’an, China; 2 Institute of Water Resources and Hydro-electric Engineering, Xi’an University of Technology, Xi’an, China; 3 Shaanxi Railway Institute, Weinan, China; National Kaohsiung University of Science and Technology / Industrial University of Ho Chi Minh, TAIWAN

## Abstract

To effectively identify the key material parameters of different zones of concrete face rockfill dams and improve the efficiency of parameter optimization, a global sensitivity analysis method of parameters based on sparse polynomial chaotic expansion (sPCE) is proposed in this paper. The latin hypercube sampling method is used to select multiple groups of material parameters, and then finite element method is used to calculate the displacement of dam characteristic nodes in dam body. On this basis, the displacement is expanded by sPCE, and the polynomial basis function is reconstructed by orthogonal matching pursuit to improve the construction and analysis efficiency of the proxy model. According to the chaos coefficients, Sobol’ indices are calculated to evaluate the influence of the material parameters and their interaction on different displacements of the dam. The results show that the sPCE model can accurately simulate dam displacement and its statistical characteristics with a relatively small sample size. The sensitivity of the same parameter has spatial variability, and under the influence of parameter levels and spatial distribution of different materials, the parameter sensitivity ranking of different zones has certain differences. The proposed method provides a new reference to sensitivity analysis and uncertainty analysis for practical engineering.

## 1 Introduction

The stress‒strain characteristics are the basis of the structural analysis of concrete face rockfill dam (CFRD). Therefore, scholars have proposed a variety of constitutive models to describe the relationship between the stress and strain of rockfill, such as the Duncan-Chang EB model [1] and elastic-plastic model [2]. These models all show the characteristics of high nonlinearity and multi-parameters, and there is an uncertainty difference between experimental material parameters and actual material parameters, which makes the analysis process costly and the uncertainty error of the results difficult to control. Therefore, it is often necessary to analyze the parameter sensitivity of the models before structural analysis [3]. Sensitivity analysis is a method to quantitatively evaluate the contribution of uncertain input variables to structural response output. Its biggest role is to identify the importance of each variable input in the model, that is, to identify important variables and secondary variables, and to filter out secondary variables in the research process to simplify the original model [4].

At present, there are many methods for parameter sensitivity analysis, which can be divided into local sensitivity analysis (LSA) and global sensitivity analysis (GSA) according to their principles. LSA is used to analyze the influence of a single parameter change on the model output variables, such as Monte Carlo sampling [5] and the first-order second-moment method [6]. It has the characteristics of simple principle and fast calculation, but the analysis results have certain limitations because the interaction between parameters is not considered. GSA can simultaneously study the influence of a single parameter change and the interaction between parameters on the uncertainty of the overall model, so it is widely used in parameter optimization of multi-parameter complex models [7, 8]. Among them, the sensitivity indices based on variance, also known as Sobol’ indices, are expressed by the ratio of variances of parameters to the total variance of model output variables, which have the characteristics of intuitive and clear statistical significance and have attracted much attention [9]. Sobol proves that if the model inputs are independent of each other, there is a unique function ANOVA decomposition of the model response relative to the uncertain input. In this method, the variance of the model response is decomposed into the sum of partial variances, which are the contribution of each parameter and the further contribution generated by the interaction of each parameter. Since then, many numerical methods have been proposed to estimate Sobol’ indices, such as the Monte Carlo method [10] and Fourier amplitude sensitivity test [11]. At present, the most effective method is to adopt a proxy model based on computer model responses. The proxy model is a statistical model used to simulate the input-output relationship of the original model, which can greatly reduce the calculation cost and improve the analysis efficiency [12, 13]. The commonly used methods of establishing a proxy model include the regression method, Kriging method, artificial neural network, etc. [14], and the polynomial chaotic expansion method (PCE) is widely used because of its concise mathematical principle, wide applicability and rapid convergence.

As a kind of the spectral approaches, PCE converts the model response into the sum of orthogonal polynomials to obtain the approximate value of the model response. In sensitivity analysis, the Parseval-Plancherel theorem can be used to analytically obtain Sobol’ indices from PCE coefficients without repeatedly running PCE as a proxy model [12, 15]. At present, PCE has been successfully applied to sensitivity analysis in different fields [12–17], and its techniques can be mainly divided into the invasive method and the non-invasive method [18]. The former is put forward under the background of the stochastic finite element method, which is used to discretize constitutive equations in physical space and random space; the latter is based on post-processing multiple analog outputs of existing numerical models [19, 20]. Compared with the invasive method, the non-invasive method is simple to use and easy to solve, so it has a wider scope of application. It should be noted that PCE easily falls into ‘dimension disaster’ in practical applications when dealing with multi-parameter and high-dimensional space problems, which leads to a significant increase in the number of expansion items and coefficients of PCE to be solved. However, sparse polynomial chaotic expansion (sPCE) based on the sparse effect criterion, established by using minimum angle regression, compressive sensing technology or other methods, can better address the ‘dimension disaster’ problem [21, 22] and reduce the calculation cost of the original model when solving the coefficients.

In this paper, a global sensitivity analysis method of parameters based on sPCE is proposed. According to this method, a proxy model of the nonlinear function relationship between the deformation of CFRDs and the material parameters of the constitutive model is established by using PCE theory, and the polynomial basis function is reconstructed by the orthogonal matching pursuit method (OMP) to solve the problem of rapid growth of the polynomial basis function in high-dimensional and high-order proxy models. Then, the global sensitivity of each parameter is accurately evaluated by Sobol’ indices. The effectiveness and rationality of this method are verified by the results of the parameter sensitivity analysis of each characteristic node of the dam body under different conditions, which provides a new reference for the uncertainty analysis of material parameters, as well as the subsequent parameter inversion and structural reliability analysis.

## 2 Methodology

### 2.1 Sparse polynomial chaotic expansion method (sPCE)

PCE was proposed by Wiener in 1938 when he studied the decomposition of a Gaussian random process. Its mathematical principle is to use the sum of orthogonal polynomials corresponding to the probability distribution type of input parameters to approximately simulate the random process [23]. After years of research and exploration, PCE has been developed and extended to simulate arbitrary random processes and is often used to establish proxy models to solve complex nonlinear problems in practical applications [24].

According to the theory of PCE, in the probability space (Ω, F, P), if the probability density function of any random variable is a quadratically integrable function, the random response model *Y* can be expanded as follows:

$$Y=a_{0}X_{0}+\sum_{i_{1}=1}^{n}a_{i_{1}}X_{1}(\xi_{i_{1}})+\sum_{i_{1}=1}^{n}\sum_{i_{2}=1}^{i_{1}}a_{i_{1}i_{2}}X_{2}(\xi_{i_{1}},\xi_{i_{2}})+\sum_{i_{1}=1}^{n}\sum_{i_{2}=1}^{i_{1}}\sum_{i_{3}=1}^{i_{2}}a_{i_{1}i_{2}i_{3}}X_{3}(\xi_{i_{1}},\xi_{i_{2}},\xi_{i_{3}})+\cdots ,$$ (1)

where $a_{0},a_{i_{1}},a_{i_{1}}{}_{i_{2}},$… are the polynomial coefficients to be solved, $\xi_{i_{1}},\xi_{i_{1}},\xi_{i_{3}},$… are random variables that are independent of each other and obey a certain probability distribution as the input variable selected in the uncertainty analysis; *X*_1_(·), *X*_2_(·), *X*_3_(·),… is the basis function of chaotic expansion. As a group of orthogonal functions, its type is determined by the probability distribution type of input variables. At present, the Askey scheme is often used to determine the basis function [25]. The total number *M* of items after expansion can be expressed as

$$M=\frac{(n+s)!}{n!s!},$$ (2)

where *n* is the number of random variables and *s* is the highest order of PCE. To meet the requirements of calculation efficiency and accuracy (convergence), fitting analysis is generally carried out by using 2~3 order polynomial expansion, and the relative error can be basically controlled within 1% [26, 27].

The determination of coefficients is the key to PCE method analysis, which directly affects the accuracy of the model output. Currently, the non-interference method is often used to calculate PCE coefficients in practice, such as the linear regression method [28, 29]. To solve the PCE coefficients based on the linear regression method, it is necessary to analyze the output variables of *N* sampling points and then calculate the undetermined coefficients by the least square method:

$$\hat{\alpha }=\arg\;\min{\sum_{i=1}^{N}[\alpha^{T}\Psi (\xi^{\left(i\right)})-Y^{\left(i\right)}]}^{2},$$ (3)

where *α* and ψ are the undetermined expansion coefficient vector and basis function matrix, respectively, *ξ*^(*i*)^ is the *i*-th sample of the input random variable vector *ξ*, and *Y*^(*i*)^ is the deterministic calculation result of the output variable under the *i*-th sample. To ensure the accuracy of the regression calculation, *N*>2*M* is generally needed.

When using the above method to calculate the PCE coefficients, the number of expansion items in the complete PCE will increase significantly with the increase in the number of input variables, which will lead to a decrease in analysis efficiency. In practical engineering analysis, the PCE model has obvious sparsity. When solving, the PCE basis function items that have great influence on the output variables can be selected, and the coefficients of the other PCE expansion items are set to 0, which not only preserves the main influence relationship between the input variables and the output variables but also reduces the number of coefficients of the expansion items to be solved, thus improving the modeling and analysis efficiency of the PCE model. Based on the sparsity of the PCE, the solution of the expansion coefficients can be expressed as:

$$\{\begin{array}{c} \hat{\alpha }=\arg\;\min\;{\left\|\alpha \right\|}_{0}\\ s.t.\;{\hat{\alpha }}^{T}\Psi (\xi^{\left(i\right)})-Y^{\left(i\right)}=0,i=1,2,\cdots ,N \end{array},$$ (4)

where ||*α*||_0_ is the number of nonzero items in the expansion coefficient vector to be determined. Selecting the basis function by Eq (4), the sPCE has the fewest nonzero coefficient expansion items, and the proxy model can still maintain high accuracy with the same or fewer input variable samples. Therefore, ensuring that the generalization error of the established proxy model is sufficiently small is the key to the selection of the basis function. At present, the commonly used methods for selecting the basis function include the convex relaxation algorithm (CR) [26] and orthogonal matching pursuit (OMP), etc. [12, 30]. Among them, OMP determines the position of the nonzero item by verifying the orthogonality between the residual and the expansion item, and then the approximate solution of the L0 norm minimization problem is directly obtained by the least square method. In this paper, OMP is used to reconstruct an important basis function to establish an sPCE proxy model.

### 2.2 Sensitivity analysis

The Sobol’ sensitivity indices based on variance decomposition are calculated from the sPCE coefficients obtained in the previous section, which can directly analyze the sensitivity of each input variable to the model output. Let the input variable be an n-dimensional independent random variable *x* = [*x*_1_,…, *x*_*n*_], and the expression of sPCE truncated at *p* can be written as

$$Y=Y_{0}+\sum_{i=1}^{p}\alpha_{i}\Psi_{i}(x_{i})+\sum_{1\leq i<j\leq p}\alpha_{i,j}\Psi_{i,j}(x_{i},x_{j})+\cdots +\alpha_{1,\cdots ,N}\Psi_{1,\cdots ,N}(x_{1},\cdots ,x_{n}).$$ (5)

The model response output is also a random variable, and its variance can be expressed as

$$D=\mathrm{Var}[Y(x)]=\int_{n}Y^{2}(x)dx-Y_{0}^{2}.$$ (6)

Due to the orthogonality of the basis function, the variance of the output response can be decomposed into the sum of the partial variance of different combinations of input variables:

$$D=\sum_{i=1}^{n}D_{i}+\sum_{1\leq i<j\leq n}D_{i,j}+,\cdots ,+D_{1,2,\cdots ,n}.$$ (7)

The partial variance is defined as $D_{i_{1},\cdots ,i_{n}}=\sum_{t\in I_{i_{1},\cdots ,i_{n}}}a_{t}^{2}$, where *a*_*t*_ is the chaotic expansion coefficient, $I_{i_{1},\cdots ,i_{n}}=\{\alpha \in (\alpha_{1},\alpha_{2},\cdots ,\alpha_{n}):\alpha_{k}=0\Leftrightarrow k\in (i_{1},\cdots ,i_{p}),\forall k=1,\cdots ,n\}$. The variance decomposition leads to a natural definition of the sensitivity indices [31]:

$$S_{i_{1},\cdots ,i_{n}}=\frac{D_{i_{1},\cdots ,i_{n}}}{D}=\sum_{t\in I_{i_{1},\cdots ,i_{n}}}\frac{a_{t}^{2}}{D}.$$ (8)

It can be seen from Formula (7) that the indices meet the following condition:

$$\sum_{i=1}^{n}S_{i}+\sum_{1\leq i<j\leq n}S_{i,j}+\cdots +S_{1,2,\cdots ,n}=1,$$ (9)

where *S*_*i*_ is the index with respect to one input variable *x*_*i*_, which is called the first-order Sobol’ index and represents the effect of *x*_*i*_ alone. $S_{i_{1},\cdots ,i_{n}}$, e.g., S_*i*,*j*_, is referred to as the higher-order Sobol’ index and is an interaction index that accounts for the effect of the interaction of the variables *x*_*i*_ and *x*_*j*_ that cannot be decomposed into the contributions of those variables separately. The total Sobol’ index of input variable *x*_*i*_ is the sum of all the Sobol’ indices involving this variable (including first-order Sobol’ index and higher-order Sobol’ index):

$$S_{i}^{T}=\sum_{\{i_{1},\ldots ,i_{n}\}⊃i}S_{i_{1},\ldots ,i_{n}}.$$ (10)

For the convenience of calculation, the total index can be written as

$$S_{i}^{T}=1-S_{∼i}$$ (11)

where *S*_~*i*_ is the sum of all sensitivity indices excluding variable *x*_*i*_.

Since the sensitivity indices can be directly obtained by algebraic operation of expansion coefficients, the main workload of sensitivity analysis based on sPCE is the deterministic analysis of the original model and the calculation of sPCE expansion coefficients, which has higher analysis efficiency than traditional Monte Carlo simulation and other methods.

## 3 The GSA of parameters based on sPCE

**Fig 1:**
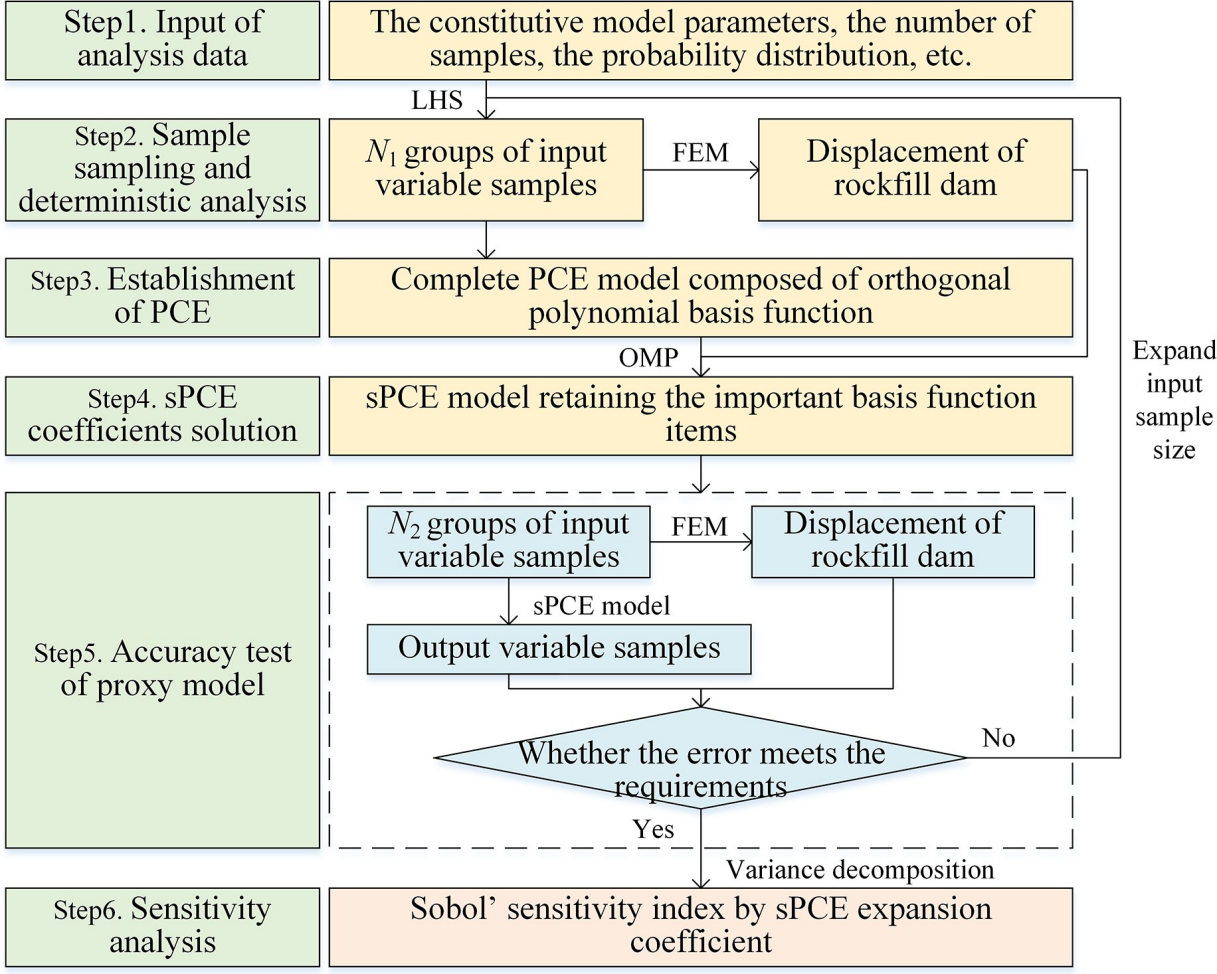
Parameter sensitivity analysis process based on sPCE.

The GSA framework of parameters based on sPCE proposed is shown in Fig 1, and the main steps are as follows:

1. Input of analysis data. The data, including the material parameters of the constitutive model, the number of parameters, the value of parameters, the number of samples and the probability distribution, are determined.
2. Sample sampling and deterministic analysis. *N*_1_ groups of input variable samples are obtained by Latin hypercube sampling (LHS), and through finite element method (FEM), the characteristic displacement of the CFRD of each sample is obtained as the output variable samples.
3. Establishment of PCE. According to the probability distribution form of the input variables, the corresponding orthogonal polynomial basis function is determined to establish the PCE model.
4. sPCE coefficient solution. According to the parameter-displacement samples obtained in step (2) and the complete PCE established in step (3), the expansion coefficients are solved by OMP, and the sPCE model is established, which retains the important basis function items.
5. Accuracy test of the proxy model. *N*_2_ groups of input variable samples are generated by LHS, and the output variable samples (dam characteristic displacement) are calculated by FEM and the sPCE proxy model. The error of the sPCE model is analyzed by comparing the calculation results, and the next sensitivity analysis can be carried out if the error is small. Otherwise, it is necessary to expand the input sample size and repeat steps (2) ~ (4).
6. Sensitivity analysis. From the sPCE expansion coefficient obtained in step (4), the Sobol’ sensitivity index of each material parameter is directly calculated by Formulas (9) ~ (11).

## 4 Case study

**Fig 2:**
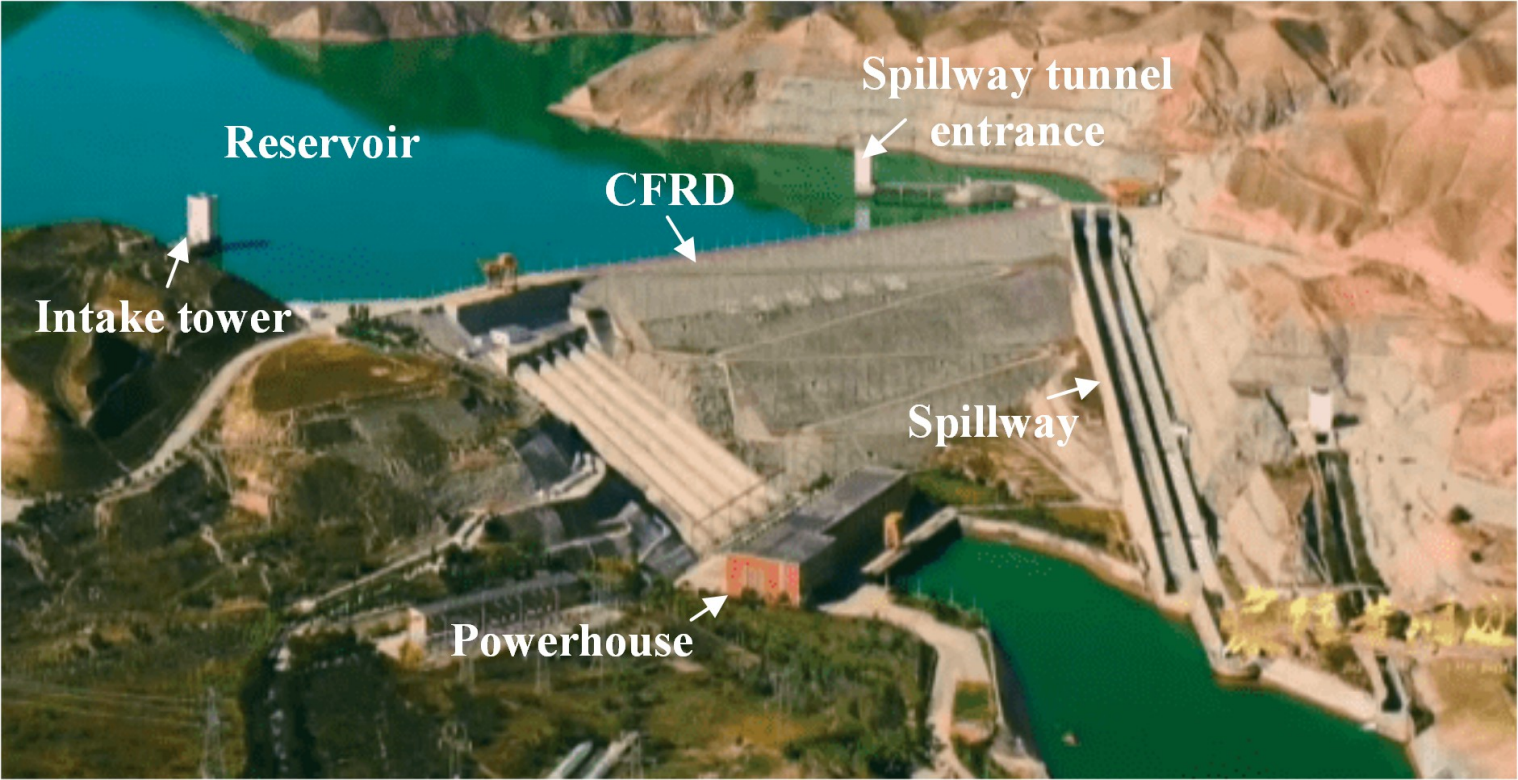
Schematic diagram of the project layout.

A large-scale cascade hydropower station used mainly for power generation, irrigation, and supplying water is located in the upper reaches of the Yellow River in Northwest China, as shown in Fig 2. The normal water level and dead water level are 2005.00 m and 1975.00 m, respectively. The main structure of the project is a CFRD, with a maximum dam height of 132.20 m. The dam crest elevation is 2010.00 m, and the dam crest length and width are 429.00 m and 10 m, respectively. The dam foundation is mostly weakly weathered schist, and the engineering geological conditions are adequate.

**Fig 3:**
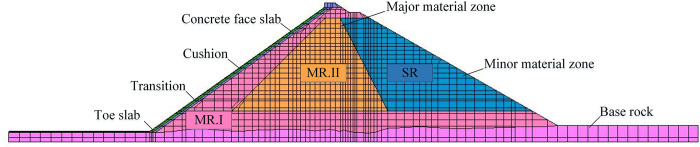
Finite element model of the CFRD.

A two-dimensional finite element model of the CFRD was established based on the typical cross section of the dam, as shown in Fig 3, which is composed of 4419 nodes and 2860 hexahedral eight-node isoparametric elements. To better simulate actual engineering, the dam foundation consolidation process, the stages of filling the dam body, and the stages of water storage are simulated in the calculation process. Then, fixed constraints are applied at the bottom of the model, and corresponding normal constraints are applied on both sides. The water pressure is applied as a surface force on the surface of the panel and the upstream foundation during the reservoir filling process.

The Duncan-Chang EB model is widely used in practical engineering because of its clear concept and easy acquisition of model parameters, so the physical and mechanical properties of geotechnical structures are described by the E-B model in this paper. The laboratory geotechnical test parameters of each material of the CFRD are shown in Table 1, in which the EB model is used for materials in the dam, such as the cushion, transition, and rockfill materials; the linear elastic constitutive model is used for the concrete structure and base rock.

**Table 1:** The laboratory geotechnical test parameters of each material.

Zones	Volume weight (kN/m^3^)	*K*	*N*	*R*_f_	*K*_ur_	*C* (kPa)	*φ* (°)	Δ*φ* (°)	*K*_b_	*m*
Cushion	21.5	1050	0.36	0.826	2100	0	49.4	8.7	1300	0.164
Transition	21.3	1090	0.34	0.89	2200	0	50.4	9.3	830	0.047
MR.I	24.8	950	0.65	0.893	1800	0	54.9	8.2	550	0.17
MR.II	22.4	1600	0.31	0.842	3000	0	58.4	6.0	800	0.03
SR	21.1	720	0.47	0.81	550	0	56.5	9.4	485	0.13
Concrete face slab, toe board			*E =* 2.17×10^4^MPa, *υ =* 0.167, *γ =* 24kN/m^3^							
Dam foundation bedrock			*E =* 2.0×10^2^MPa, *υ =* 0.25, *γ =* 30kN/m^3^							

### 4.1 Parameter sensitivity analysis of the EB model

#### 4.1.1 Establishment and verification of sPCE

When only analyzing the parameter sensitivity of the EB model, the influence of different material zones of rockfill should be excluded. Therefore, it is necessary to assume that the CFRD is a homogeneous dam with a single material, and the mechanical parameters of rockfill refer to the major material zone (MR.I). Furthermore, the parameters of rockfill are assumed to obey a Gaussian distribution, in which the mean values are the parameter values of the laboratory geotechnical test, with a coefficient of variation of 0.1. The statistical eigenvalue and sampling range of each parameter of the EB model are shown in Table 2.

**Table 2:** The statistical eigenvalue and sampling range.

Parameter	Statistical eigenvalue		Range
	*Μ*	*σ*	
*K*	950	95	[665,1235]
*K*_*b*_	550	55	[385,715]
*R*_*f*_	0.893	0.0893	[0.6251,1.1609]
*K*_*ur*_	1800	180	[1260,2340]
*φ*_0_ (°)	54.9	5.49	[38.43,71.37]
Δ*φ* (°)	8.2	0.82	[5.74,10.66]
*n*	0.65	0.065	[0.455,0.845]
*m*	0.17	0.017	[0.119,0.221]

LHS is used to randomly sample the parameters of the EB model, and 400 groups of different parameter combination data are generated. Then, 400 groups of dam displacement calculation results are obtained by FEM. 400 groups of parameter-displacement data are divided into fitting samples and verification samples. According to the theory of sPCE, regression fitting and verification analysis of 8-dimensional 2nd-order sPCE are carried out with the EB model parameters as the model input and dam displacement as the model output.

**Fig 4:**
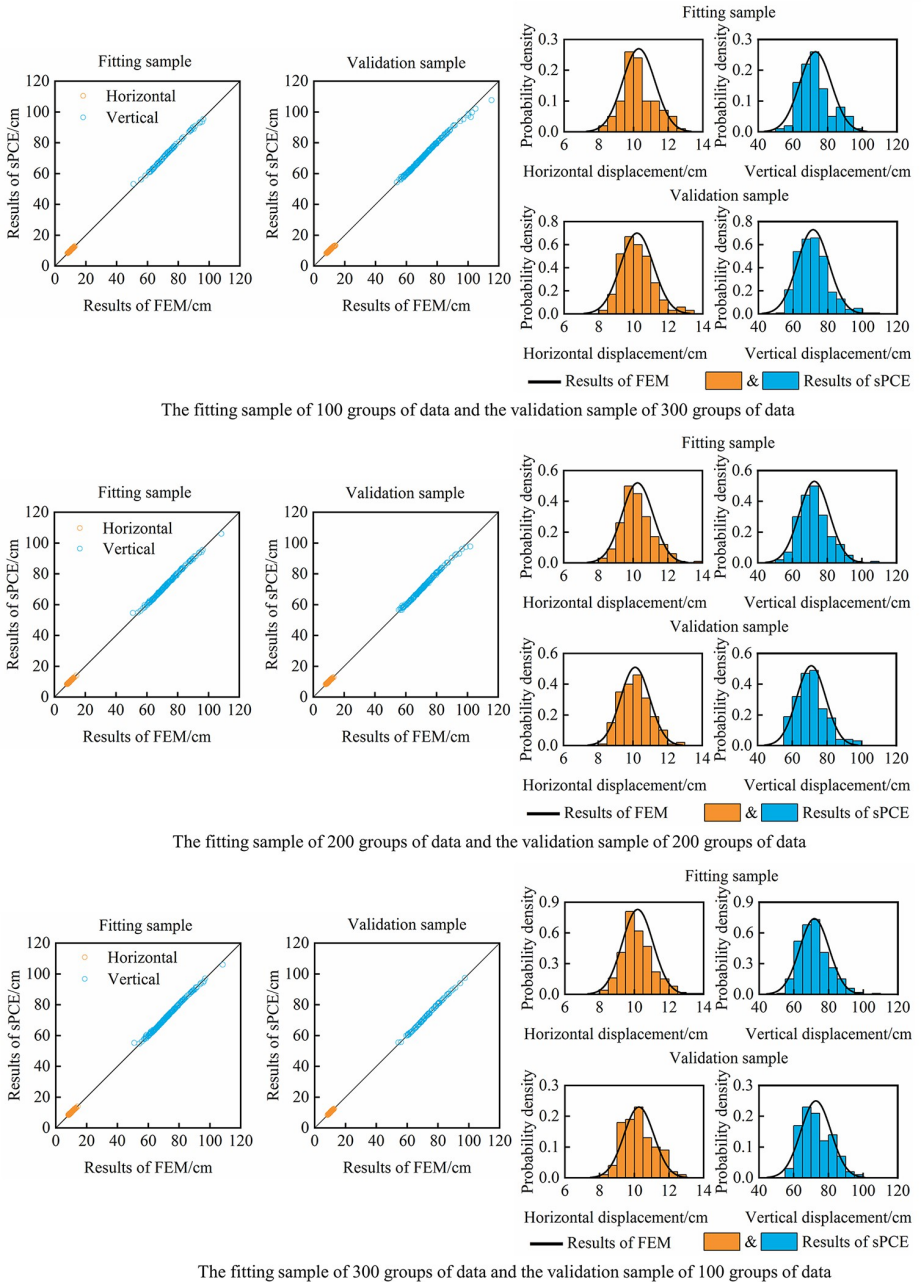
Comparison between the FEM results and the sPCE model response.

To show the analysis effect more intuitively, the sPCE model responses and the FEM results of the maximum vertical displacement and horizontal displacement at this position of the dam are calculated, and the scatter plot and comparison of the probability distribution are shown in Fig 4. The following qualitative analysis conclusions can be drawn: (1) The sPCE model has high simulation accuracy, and the vertical displacement and horizontal displacement of the dam obtained by the sPCE model are basically the same as those calculated by FEM, and all points in the figure are located on the ‘Displacement by FEM = Displacement by sPCE’ line except for a few displacement values. (2) For different samples, the calculated results of vertical displacement and horizontal displacement of the dam obey Gaussian distribution, and the probability distribution of sPCE model response value is basically the same as that of FEM result. (3) Comparing the fitting and verification effects in different sample sizes, the sample size has little influence on the analysis accuracy of sPCE model, and sPCE model still has a high simulation effect in the small sample of 100 groups of data.

In addition, the root mean square percentage error (RMSPE), mean absolute percentage error (MAPE) and correlation coefficient (R) are selected as evaluation indices to better evaluate the accuracy and applicability of the sPCE model by analyzing the evaluation indices and comparing the displacement probability distribution, as shown in Tables 3 and 4. (1) In the three fitting samples, the RMSPE and MAPE of the vertical displacement and horizontal displacement of the sPCE model are lower than 2.5×10^−4^ and 1.2×10^−5^, respectively. In different verification samples, the RMSPE and MAPE of the vertical displacement and horizontal displacement are below 3.1×10^−4^ and 1.4×10^−5^, respectively. The correlation coefficient of each sample is not less than 0.997. (2) Comparing the analysis errors in different samples, with the increase in fitting sample size, the sPCE model can better obtain the nonlinear relationship between parameters and displacements from samples, reducing the fitting errors of vertical displacement and horizontal displacement. As the fitting sample is increased by two times (100 to 300 groups), the analysis error only decreases slightly, basically in the same order of magnitude, indicating that the fitting effects did not differ significantly. In different verification samples, the analysis error increases with increasing sample size, but the error changes slightly, which also includes the influence of different fitting sample sizes. That is, when the fitting sample size is the same, the analysis error change of the validation sample will be smaller. (3) The error of the probability distribution of vertical displacement and horizontal displacement obtained by the sPCE model is at a low level, in which the error of the mean value is less than 0.12% and the error of the standard deviation is less than 2.06%. The error of the standard deviation in the verification sample is slightly larger than that in the fitting sample, and there is no obvious trend change in the error of the probability distribution under different sample sizes.

**Table 3:** The calculation error of the sPCE model.

Displace-ment	Fitting sample size	Evaluation index			Validation sample size	Evaluation index		
		RMSPE	MAPE	R		RMSPE	MAPE	R
Vertical	100	2.47×10^−4^	1.11×10^−5^	0.999	300	2.93×10^−4^	1.26×10^−5^	0.998
	200	2.06×10^−4^	5.72×10^−6^	0.998	200	2.21×10^−4^	6.43×10^−6^	0.998
	300	1.61×10^−4^	3.68×10^−6^	0.998	100	1.98×10^−4^	4.07×10^−6^	0.998
Horizon-tal	100	2.45×10^−4^	1.09×10^−5^	0.998	300	3.01×10^−4^	1.36×10^−5^	0.997
	200	2.03×10^−4^	6.17×10^−6^	0.998	200	2.18×10^−4^	7.00×10^−6^	0.997
	300	1.63×10^−4^	3.97×10^−6^	0.998	100	1.90×10^−4^	4.52×10^−6^	0.998

**Table 4:** The probability distribution error of the sPCE model response.

Displacement	Fitting sample size	Relative error/%		Validation sample size	Relative error/%	
		*Μ*	*σ*		*μ*	*σ*
Vertical	100	0.03	0.39	300	0.10	1.44
	200	0.05	0.26	200	0.04	0.49
	300	0.04	0.69	100	0.07	2.05
Horizontal	100	0.03	0.16	300	0.11	1.82
	200	0.06	0.34	200	0.03	0.64
	300	0.03	0.60	100	0.05	1.93

The above conclusions show that sPCE has high simulation accuracy and good adaptability to the E-B model, which can obtain relatively accurate dam displacement and its statistical characteristics under small samples. Therefore, it is feasible to establish a proxy model based on sPCE for the parameter sensitivity analysis of the E-B model.

#### 4.1.2 Parameter sensitivity analysis

**Fig 5:**
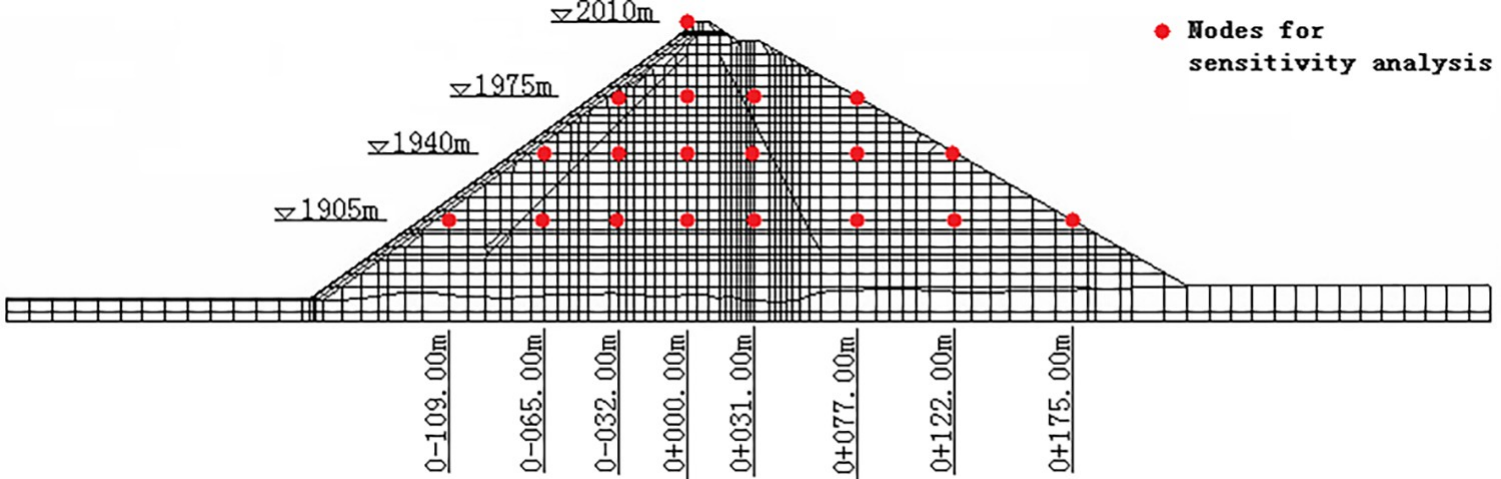
Node location selected for sensitivity analysis.

To fully reflect the influence of the E-B model parameters on the displacement of different parts of the dam body, a total of 19 nodes in different parts of the typical cross section of the dam are selected for parameter sensitivity analysis. The position of each node is shown in Fig 5. with 1 node located at the dam crest, 3 nodes in MR.I, 8 nodes in MR.II, and 7 nodes in SR. The horizontal distance of each node is 31~53m, and the vertical distance is 35m, evenly distributed to ensure its representativeness. The determination of parameter sensitivity includes the following steps: (1) The sPCE model is established for the displacement value of each node, and the parameters of the E-B model are sorted according to the calculated sensitivity index. (2) Summarizing and counting the parameter sensitivity ranking of each node, the proportion of sensitivity ranking of each parameter to the total statistical node is analyzed to determine the overall sensitivity of the dam body to each parameter.

**Fig 6:**
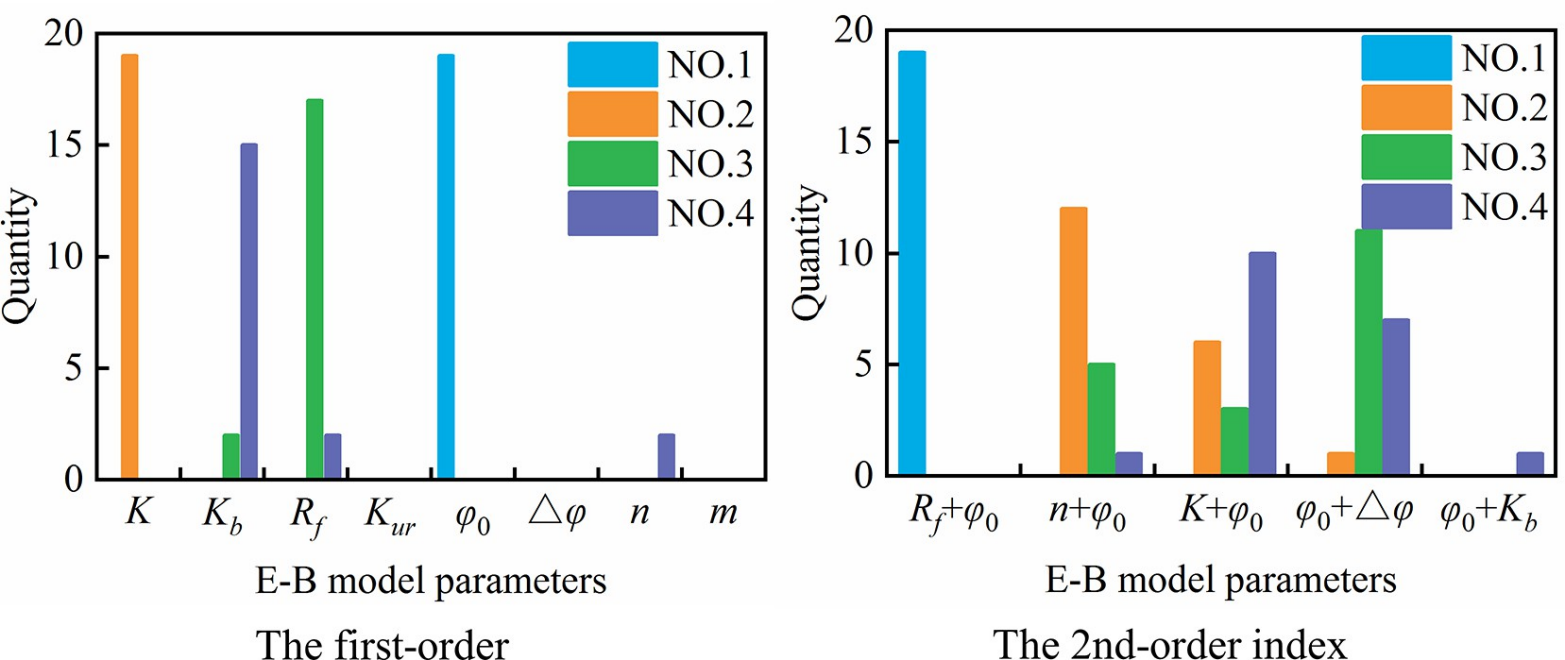
Parameter sensitivity statistics of vertical displacement.

**Fig 7:**
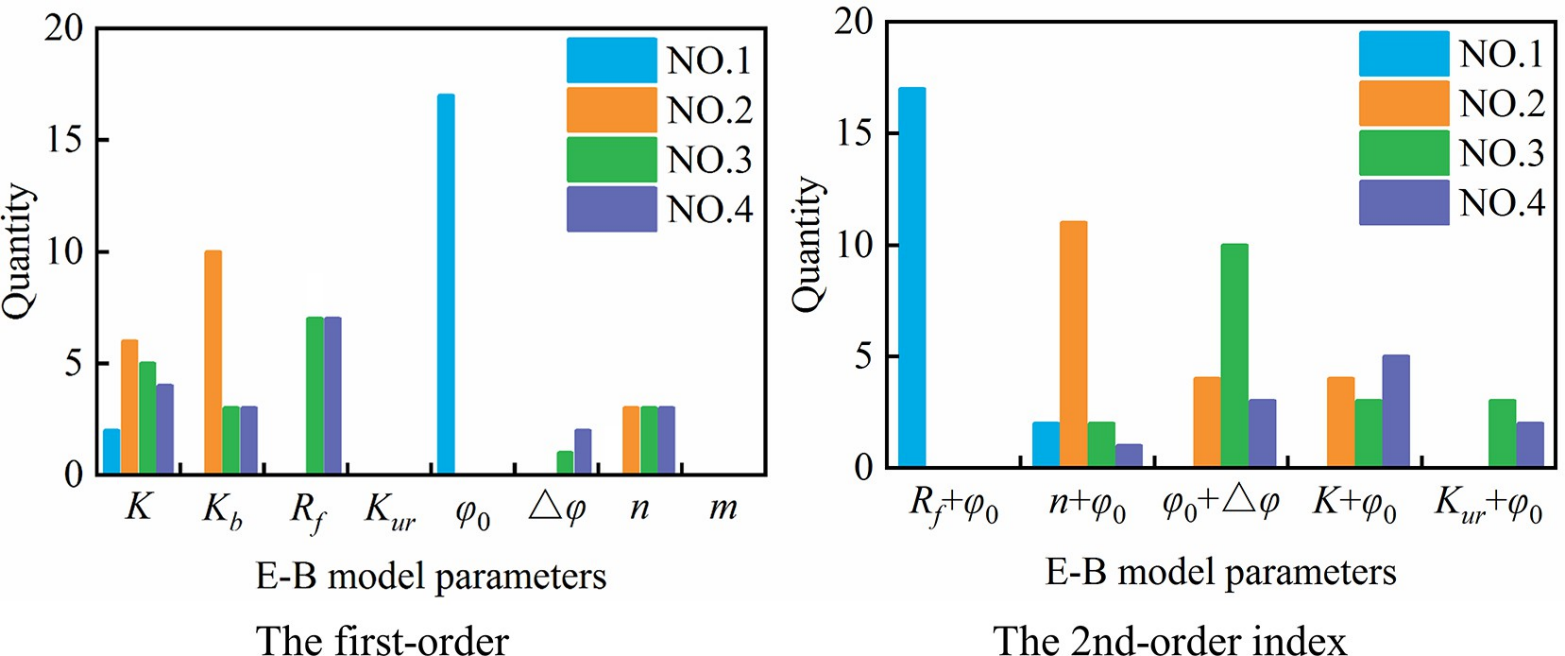
Parameter sensitivity statistics of horizontal displacement.

Figs 6 and 7 count the number of times that each parameter accounts for the top 4 in sensitivity of each node under vertical displacement and horizontal displacement, respectively. As seen from the figures, *φ*_0_ and *K* rank 1st and 2nd, respectively, in the vertical displacement parameter sensitivity of all 19 nodes. The sensitivity of *R*_*f*_ ranks 3rd in 89.5% of the nodes, that of *K*_*b*_ ranks 4th in 78.9% of the nodes, and *n* ranks 4th in sensitivity only among 10.5% of the nodes. The sensitivities of *K*_*ur*_, △*φ* and *m* in all nodes are not in the top four. For parameter interaction, *R*_*f*_ + *φ*_0_, *n* + *φ*_0_, *K* + *φ*_0_ and *φ*_0_ + △*φ* rank in the top four in almost all nodes. For horizontal displacement, the sensitivity ranking of the parameters is more complicated and diverse than that of vertical displacement. *φ*_0_ and *K* rank 1st in sensitivity among 89.5% and 10.5% of nodes, respectively, while *K*, *K*_*b*_ and *n* rank 2nd in sensitivity among 31.6%, 52.6% and 15.8% of nodes, respectively, and 3rd to 4th in sensitivity among some nodes. In addition, the sensitivities of *R*_*f*_ and △*φ* also exist in the 3rd to 4th places of some nodes. For parameter interaction, similar to vertical displacement, *R*_*f*_ + *φ*_0_, *n* + *φ*_0_, *K* + *φ*_0_ and *φ*_0_ + △*φ* also rank in the top four in most nodes.

The above conclusions show that the sensitivity of displacement in different parts of the dam body to the parameters of the EB model is different. When the displacement of an individual node of the dam body is selected as the target for parameter sensitivity analysis, the analysis conclusion may vary with the spatial position of the sample points, and the obtained parameter sensitivity results have certain limitations.

The total index of parameter sensitivity of the above nodes is counted, and the average sensitivity index of each parameter is calculated as shown in Table 5. According to the average sensitivity index, the order of parameter sensitivity of vertical displacement is *φ*_0_ > *K* > *R*_*f*_ > *K*_*b*_ > *n* > △*φ* > *K*_*ur*_ > *m*, and the order of parameter sensitivity of horizontal displacement is *φ*_0_ > *K* > *K*_*b*_ > *n* > *R*_*f*_ > △*φ* > *K*_*ur*_ > *m*. Generally, dam displacement is highly sensitive to *φ*_0_, sensitive to *K*, *R*_*f*_, *n* and *K*_*b*_, and weak to △*φ*, *K*_*ur*_ and *m*. This conclusion is basically the same as that obtained by orthogonal analysis and the Morris method [32–35].

**Table 5:** Average sensitivity index of each parameter.

Displacement	*K*	*K*_*b*_	*R*_*f*_	*K*_*ur*_	*φ*_0_	△*φ*	*n*	*m*
Vertical	1.17×10^−1^	5.22×10^−2^	6.01×10^−2^	2.54×10^−4^	7.52×10^−1^	6.13×10^−3^	2.21×10^−2^	2.26×10^−4^
Horizontal	1.30×10^−1^	1.06×10^−1^	5.86×10^−2^	5.25×10^−3^	6.28×10^−1^	1.77×10^−2^	6.59×10^−2^	3.03×10^−3^

**Fig 8:**
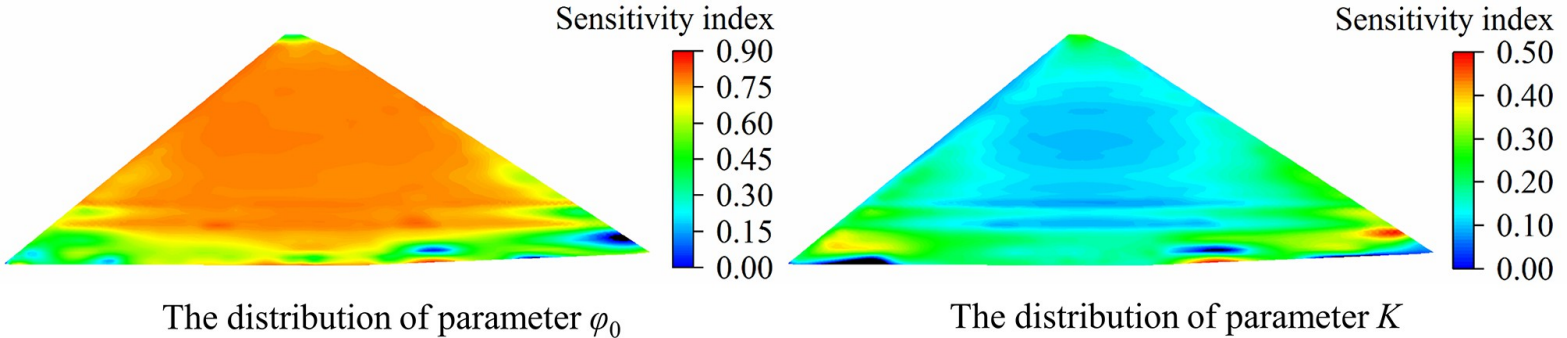
The sensitivity index distribution of vertical displacement.

**Fig 9:**
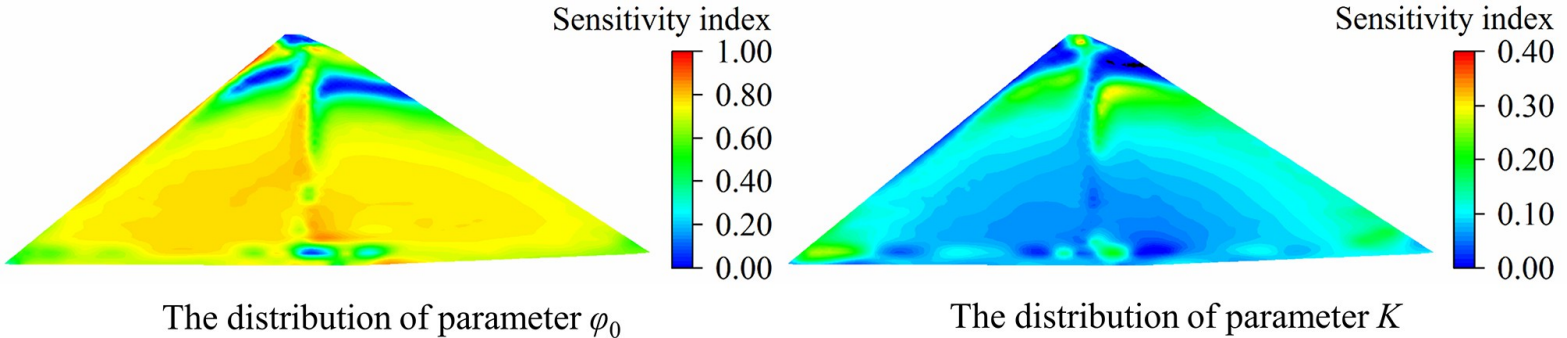
The sensitivity index distribution of horizontal displacement.

Additionally, to more intuitively show the spatial difference in parameter sensitivity, parameter sensitivity analysis is conducted on the displacements of all nodes on the typical cross section of the dam, and the analysis results are shown in S1 and S2 Tables. With parameters *φ*_0_ and *K* as examples, the sensitivity index distribution (including the part of the rockfill body only, excluding foundation and concrete structures such as face slab) is plotted as shown in Figs 8 and 9. The following conclusions can be drawn: (1) The vertical displacement of most parts of the dam body is highly sensitive to *φ*_0_, and only the parts located at the dam crest, upstream and downstream dam toe and near the dam foundation have low sensitivity to *φ*_0_. The sensitivity index distribution of parameter *K* is basically opposite to that of parameter *φ*_0_. The vertical displacement near the dam center and the upstream panel cushion is less sensitive to *K*, and the parts located at the upstream and downstream dam toe and near the dam foundation are relatively sensitive to *K*. (2) For horizontal displacement, the parts with high sensitivity to parameter *φ*_0_ are concentrated near the 1/10~2/3 dam height in the middle of the dam body and near the upstream face slab cushion. The parts near the dam crest and 4/5 dam height have low sensitivity to *φ*_0_. The sensitivity of the displacement of the upstream and downstream dam slopes near the dam crest and the position near the dam foundation to the parameter *K* is low, while the parts located at the 4/5 dam height and near the upstream and downstream dam toe are relatively sensitive to the parameter *K*.

### 4.2 Parameter sensitivity analysis of different zones

#### 4.2.1 Establishment and verification of sPCE

Based on the analysis results in Section 4.1, five parameters with relatively high sensitivity, namely, *φ*_0_, *K*, *R*_*f*_, *K*_*b*_ and *n*, are selected as the research objects to analyze the parameter sensitivity of dam material in different zones. Similar to the previous analysis, the parameters of rockfill are also assumed to obey Gaussian distribution, in which the mean values are the parameter values of the laboratory geotechnical test, with a coefficient of variation of 0.1. The statistical eigenvalue and sampling range of each parameter of the EB model in different zones are shown in Table 6.

**Table 6:** The statistical eigenvalue and sampling range.

Zone	Parameter	Statistical eigenvalue		Range
		*Μ*	*σ*	
MR.I	*K*	950	95	[665,1235]
	*K*_*b*_	550	55	[385,715]
	*R*_f_	0.893	0.0893	[0.625,1.161]
	*φ*_0_°)	54.9	5.49	[38.43,71.35]
	*n*	0.65	0.065	[0.455,0.845]
MR.II	*K*	1600	160	[1120,2080]
	*K*_*b*_	800	80	[560,1040]
	*R*_f_	0.842	0.0842	[0.589,1.095]
	*φ*_0_°)	58.4	5.84	[40.88,75.92]
	*n*	0.31	0.031	[0.217,0.403]
SR	*K*	720	72	[504,936]
	*K*_*b*_	485	48.5	[339.5,630.5]
	*R*_f_	0.81	0.081	[0.567,1.053]
	*φ*_0_°)	56.5	5.65	[39.55,73.45]
	*n*	0.47	0.047	[0.329,0.611]

**Fig 10:**
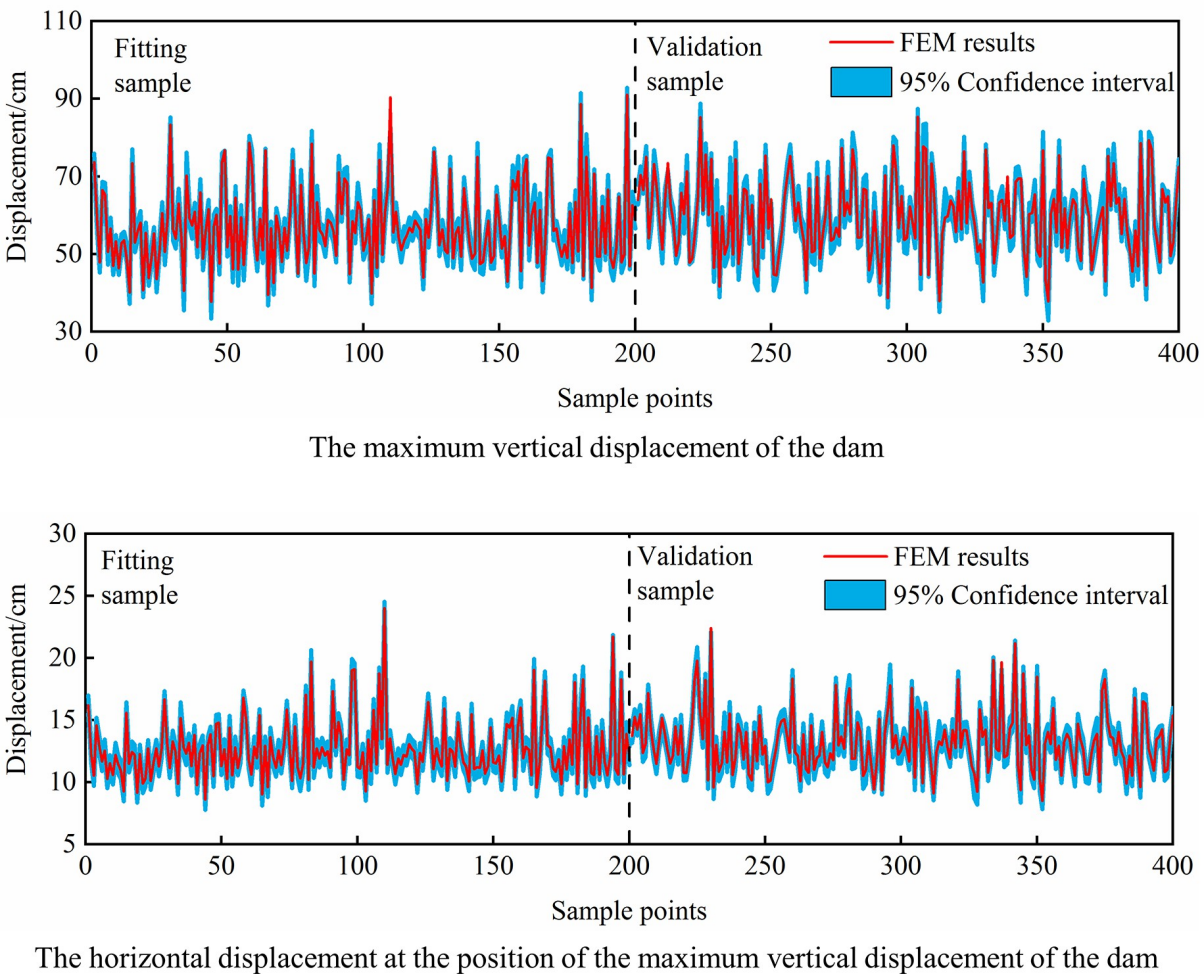
The FEM results and the displacement interval of the sPCE model response.

**Fig 11:**
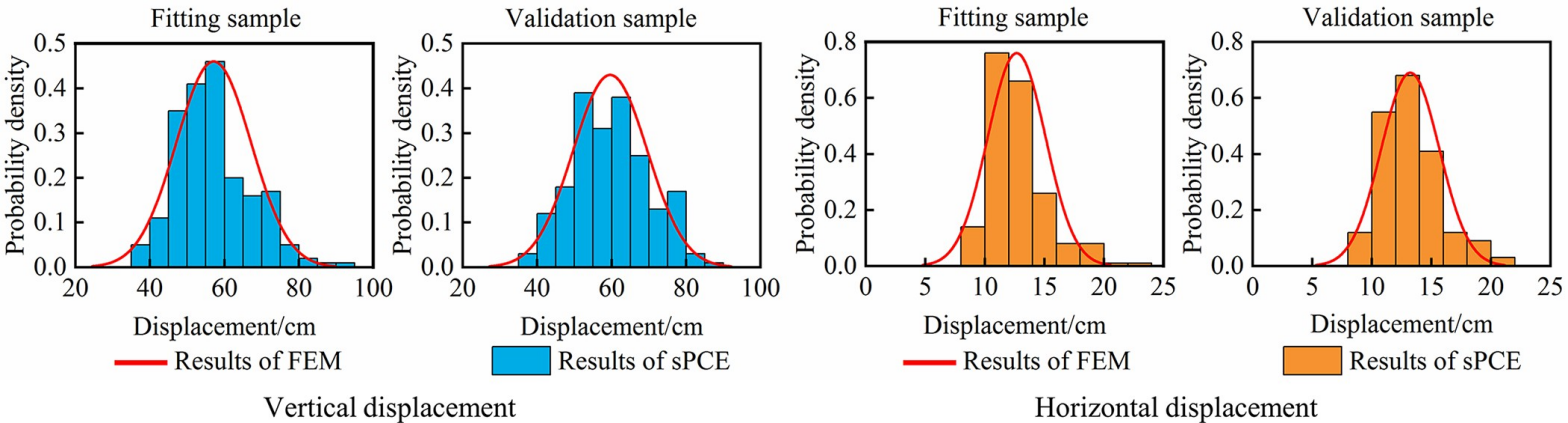
The probability distribution of dam displacement.

The analysis results in Section 4.1 show that the PEC method has high calculation accuracy for different sample sizes. LHS is used to generate 400 groups of different parameter combination data, and 400 groups of dam displacement calculation results are obtained by FEM. Then, the 200 groups of parameter-displacement data are used as fitting samples, and the remaining 200 groups of data are used as verification samples. According to the theory of sPCE, regression fitting and verification analysis of 15-dimensional 2nd-order sPCE are carried out with the EB model parameters of different zones as the model input and dam displacement as the model output. Fig 10 shows the dam displacement values by FEM and the confidence interval of the sPCE model response at the 95% confidence level, and the probability distribution of dam displacement is shown in Fig 11. The following conclusions can be drawn: (1) The sPCE model has a good simulation effect on dam displacement. There are no obvious deviation errors between the displacements calculated by FEM and the responses of the sPCE model in the fitting sample and verification sample, and most of the calculated values of vertical displacement and horizontal displacement are within the confidence interval. Only 17 groups of vertical displacement values and 6 groups of horizontal displacement values are outside the confidence interval, accounting for 4.25% and 1.5% of the total sample, respectively. (2) In the probability distribution, the calculated results of the vertical displacement and horizontal displacement of the dam by the FEM and sPCE model still obey a Gaussian distribution, and the probability distribution of the displacement simulated by the sPCE model is basically the same as that calculated by the FEM.

The calculation errors of the sPCE model are shown in Tables 7 and 8. (1) The simulation error of the sPCE model is small, the RMSPE and MAPE of the vertical displacement and horizontal displacement are less than 1×10^−3^, and the correlation coefficients of the results are greater than 0.97. Compared with the fitting sample, the simulation error of the verification sample increases slightly, being basically on the same order of magnitude as that of the fitting sample. (2) The sPCE model is relatively accurate in simulating the probability distribution of each displacement, in which the error of the mean value is less than 0.2%, the error of the standard deviation is less than 4.13%, and the errors of the mean value and standard deviation in the verification sample are slightly larger than those in the fitting sample.

**Table 7:** The calculation error of the sPCE model.

Displace-ment	Fitting sample size	Evaluation index			Validation sample size	Evaluation index		
		RMSPE	MAPE	R		RMSPE	MAPE	R
Vertical	200	3.06×10^−4^	9.32×10^−6^	0.998	200	3.25×10^−4^	9.57×10^−6^	0.997
Horizontal	200	3.55×10^−4^	1.11×10^−5^	0.997	200	7.25×10^−4^	8.02×10^−6^	0.972

**Table 8:** The probability distribution error of the sPCE model response.

Displacement	Fitting sample size	Relative error/%		Validation sample size	Relative error/%	
		*Μ*	*σ*		*μ*	*σ*
Vertical	200	0.05	0.42	200	0.20	4.13
Horizontal	200	0.03	0.13	200	0.07	0.47

The above conclusions show that sPCE still has high simulation accuracy for dam displacement when there are different material zones in the dam body, so it can be used for sensitivity analysis of rockfill material parameters in different zones.

#### 4.2.2 Parameter sensitivity analysis

**Fig 12:**
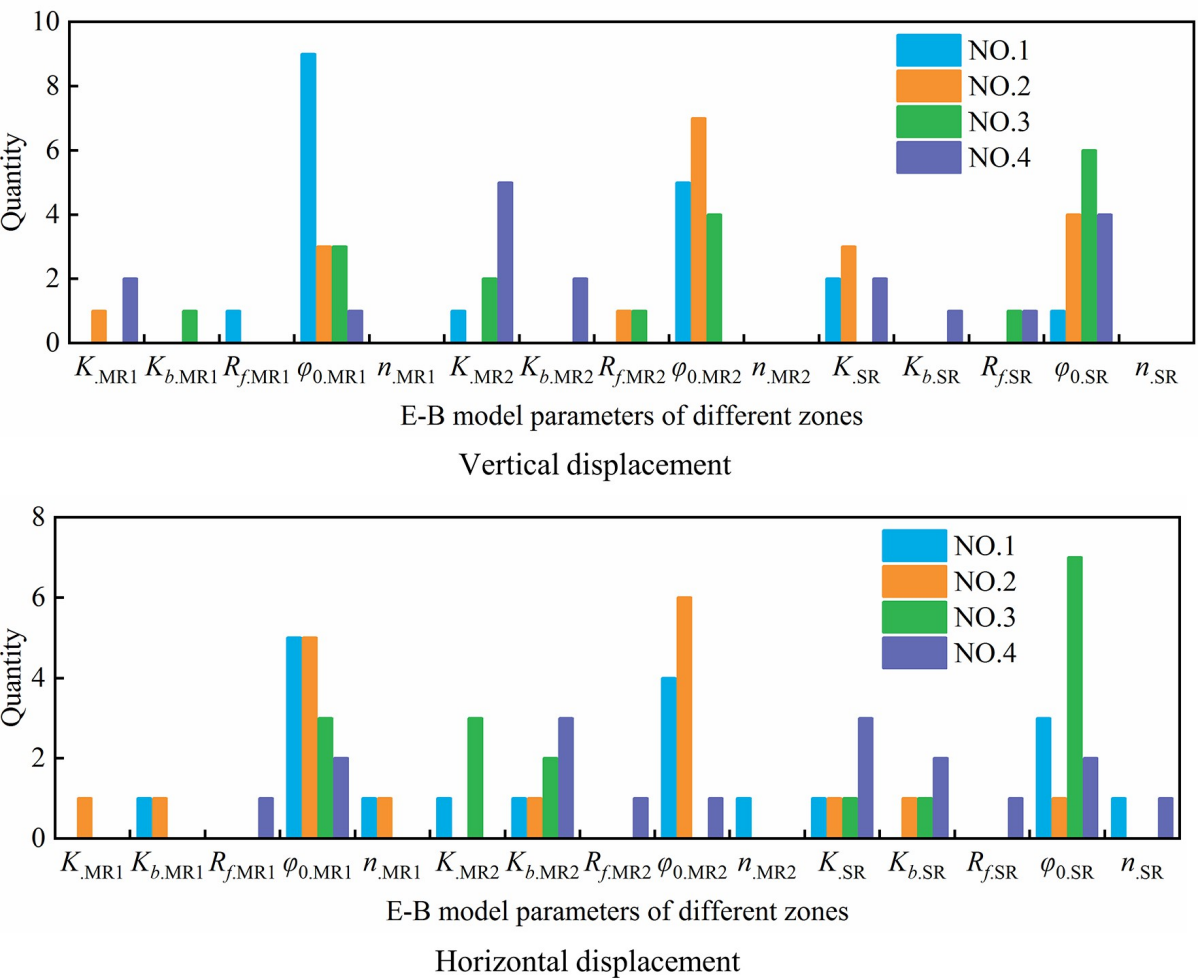
First-order sensitivity index statistics of single parameter in different zones.

**Fig 13:**
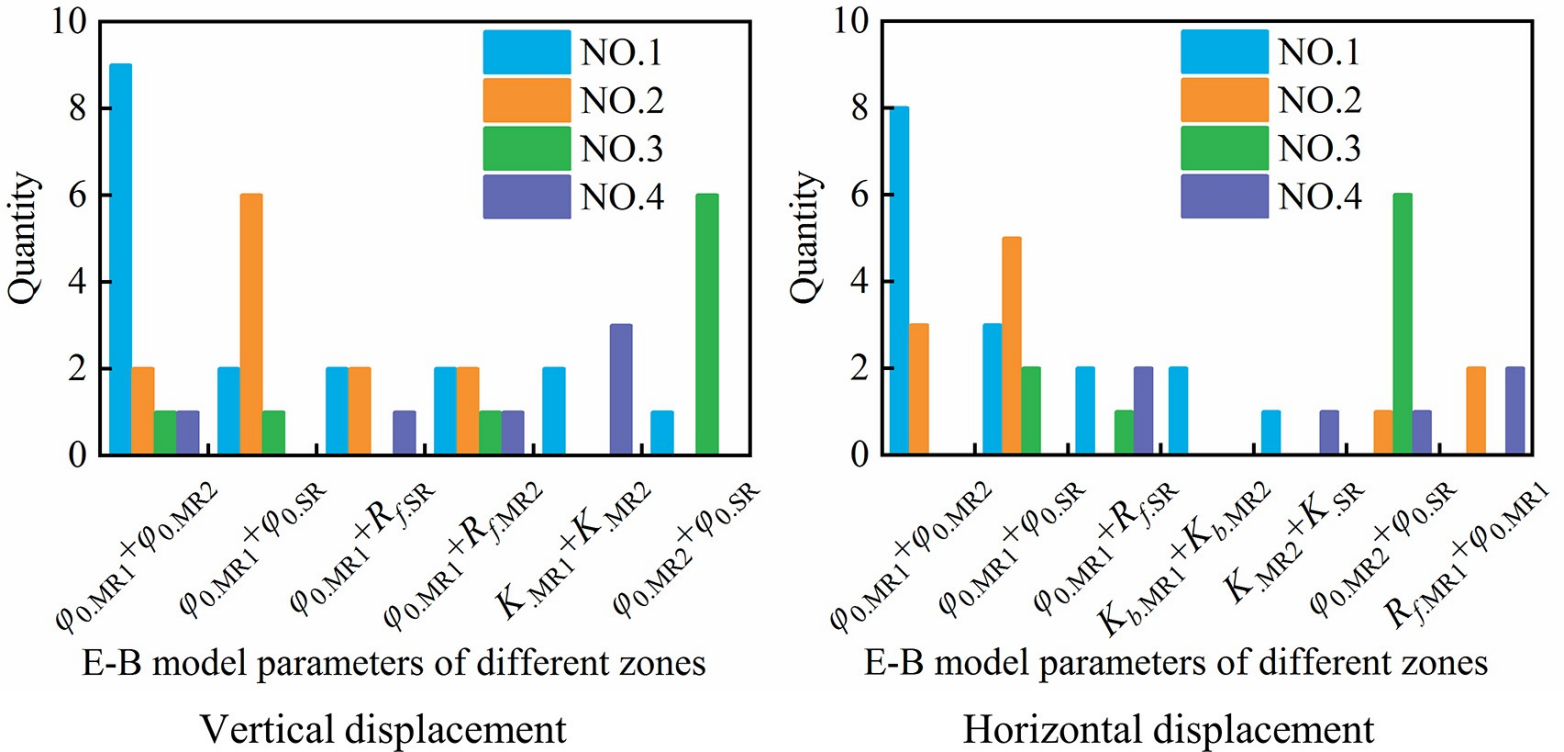
Second-order sensitivity index statistics of parameter interaction in different zones.

To fully reflect the influence of the E-B model parameters in different zones on the displacement of different parts of the dam body, a total of 19 nodes in different parts of the typical cross section of the dam body, which are the same as in Fig 5, are also selected for parameter sensitivity analysis. Similar to the analysis steps in Section 4.1.2, the sensitivity index of each parameter is calculated and sorted, and Figs 12 and 13 count the number of times that each parameter (or parameter interaction) in different zones accounts for the top 4 in sensitivity of each node under vertical displacement and horizontal displacement, respectively.

From these figures, we see that *φ*_0.MR1_, *φ*_0.MR2_ and *φ*_0.SR_ rank in the top three in the vertical displacement parameter sensitivity of more than 78.9% of nodes, and the sensitivities of *K*_.MR1_, *K*_.MR2_ and *K*_.SR_ rank 2nd to 4th in some nodes. The sensitivities of *K*_*b*_ and *R*_*f*_ of different zones only rank in the top four in vertical displacement of few nodes, and sensitivities of *n*._MR1_, *n*._MR2_ and *n*._SR_ in all nodes are not in the top four. For parameter interaction, *φ*_0.MR1_+*φ*_0.MR2_ ranks in the top four among 68.4% of nodes, and the interactions between *φ*_0.MR1_ and parameters *φ*_0.SR_, *R*_*f*.SR_ and *R*_*f*.MR2_ also rank in the top in some nodes. For horizontal displacement, the sensitivity ranking is more diverse than that of vertical displacement. *φ*_0.MR1_、*φ*_0.MR2_ and *φ*_0.SR_ rank in the top three in the parameter sensitivity of more than 57.9% of nodes, and *K*_.MR2_, *K*_*b*.MR2_, *K*_.SR_ and *K*_*b*.SR_ rank in the top four in sensitivity among 21.1%, 36.8%, 31.6% and 21.1% of nodes, respectively. The sensitivities of *K*_.MR1_, *K*_*b*.MR1_, *n*_.MR1_, *n*_.MR2_ and *n*_.SR_ rank 1st to 2nd in respective nodes, while the sensitivities of *R*_*f*_ of different zones are not in the top three in all nodes. For parameter interaction, similar to vertical displacement, *φ*_0.MR1_ has a great influence on the parameter interaction, and the interaction between *φ*_0.MR1_ and *φ*_0.MR2_ ranks in the top two in parameter sensitivity of 57.9% nodes, and the interaction between *φ*_0.MR1_ and *φ*_0.SR_, *R*_*f*.*SR*_ and *R*_*f*.*MR*2_ ranks 1st to 4th in some nodes.

According to the total index of parameter sensitivity, the average sensitivity index of each parameter in different zones is calculated as shown in Table 9. Comparing the average sensitivity index, the order of parameter sensitivity of vertical displacement is *φ*_0.MR1_ > *φ*_0.MR2_ > *φ*_0.SR_ > *K*_.SR_ > *K*_.MR2_ > *K*_.MR1_ > *R*_*f*.SR_ > *R*_*f*.MR2_ > *R*_*f*.MR1_ > *K*_*b*.MR1_ > *K*_*b*.MR2_ > *K*_*b*.SR_ > *n*_.MR2_ > *n*_.MR1_ > *n*_.SR_, and the order of parameter sensitivity of horizontal displacement is *φ*_0.MR1_ > *φ*_0.SR_ > *φ*_0.MR2_ > *K*_.MR1_ > *n*_.MR2_ > *K*_.MR2_ > *K*_*b*.MR2_ > *K*_.SR_ > *K*_*b*.SR_ > *K*_*b*.MR1_ > *n*_.SR_ > *n*_.MR1_ > *R*_*f*.MR2_ > *R*_*f*.MR1_ > *R*_*f*.SR_.

**Table 9:** Average sensitivity index of each parameter in different zones.

Displacement	Average sensitivity index				
Vertical	*K*_.MR1_	*K*_*b*.MR1_	*R*_*f*.MR1_	*φ*_0.MR1_	*n*_.MR1_
	5.73×10^−2^	7.12×10^−3^	1.94×10^−2^	4.45×10^−1^	1.57×10^−4^
	*K*_.MR2_	*K*_*b*.MR2_	*R*_*f*.MR2_	*φ*_0.MR2_	*n*_.MR2_
	7.02×10^−2^	6.01×10^−3^	2.27×10^−2^	3.36×10^−1^	3.38×10^−4^
	*K*_.SR_	*K*_*b*.SR_	*R*_*f*.SR_	*φ*_0.SR_	*n*_.SR_
	7.72×10^−2^	3.53×10^−3^	2.57×10^−2^	2.44×10^−1^	3.89×10^−5^
Horizontal	*K*_.MR1_	*K*_*b*.MR1_	*R*_*f*.MR1_	*φ*_0.MR1_	*n*_.MR1_
	9.10×10^−2^	2.09×10^−2^	5.49×10^−3^	3.37×10^−1^	1.72×10^−2^
	*K*_.MR2_	*K*_*b*.MR2_	*R*_*f*.MR2_	*φ*_0.MR2_	*n*_.MR2_
	5.85×10^−2^	5.17×10^−2^	6.85×10^−3^	2.59×10^−1^	5.98×10^−2^
	*K*_.SR_	*K*_*b*.SR_	*R*_*f*.SR_	*φ*_0.SR_	*n*_.SR_
	4.33×10^−2^	3.50×10^−2^	3.70×10^−3^	2.69×10^−1^	2.08×10^−2^

Compared with the results of parameter sensitivity analysis under a single material in Section 4.1.2, the following conclusions can be drawn about parameter sensitivity: (1) The overall ranking of parameter sensitivity in different zones of vertical displacement is *φ*_0_ > *K* > *R*_*f*_ > *K*_*b*_ > *n*, which is the same as that under a single material. For the same kind of parameters of different material zones, such as *φ*_0.MR1_, *φ*_0.MR2_ and *φ*_0.SR_, there is no obvious rule for its sensitivity ranking. (2) For horizontal displacement, the sensitivity ranking of different material parameters is generally similar to that of a single material, but there are also some differences. Except for *φ*_0_ and *R*_*f*_, the parameter sensitivity of *K*, *K*_*b*_ and *n* of each zone is not significantly different, and the sensitivity ranking of *K*, *K*_*b*_ and *n* of each zone is not in complete accord. The overall ranking of parameter sensitivity is *φ*_0_ > (*K*、*K*_*b*_、*n*) > *R*_*f*_. Therefore, the difference in the parameter level and material zoning is the reason for the difference in the parameter sensitivity analysis results, but the overall law is not much different. Although the overall rule of parameter sensitivity may not be different, the specific project should be analyzed specifically, such as the sensitivity ranking of parameters *K*, *K*_*b*_ and *n* in the EB model.

## 5 Conclusion

In this paper, a global sensitivity analysis method of parameters based on sPCE is proposed. By quantifying the contribution of each input variable to the model response variance through Sobol’ index, the uncertainty of the influence of various material parameters and their interactions in the constitutive model of CFRDs on dam displacement is comprehensively reflected. Taking the EB model commonly used in engineering as an example, the sensitivity of the displacement of a CFRD to various parameters is accurately analyzed, and the main conclusions are as follows:

1. In the two cases of single material and multiple material zones, the simulation accuracy of sPCE for vertical displacement and horizontal displacement of the dam body maintains a high level. The RMSPE and MAPE of the sPCE model are less than 1.0×10^−3^ and 1.5×10^−5^, respectively, and the correlation coefficient is greater than 0.97, which shows that this method has high reliability and authenticity in the process of parameter sensitivity analysis.
2. There are spatial differences in the sensitivity of dam displacement to the material parameters; that is, the sensitivity of displacement in different parts to the same parameter is different, and the sensitivity of displacement in different directions to the same parameter is also different. For example, the position with high sensitivity of vertical displacement to *φ*_0_ is located in most areas in the middle of the dam body, and the area with high sensitivity of horizontal displacement to *φ*_0_ is located in the middle and lower parts of the dam body. Therefore, the displacement in the area with high sensitivity to parameters should be selected to improve the accuracy of analysis in parameter inversion analysis. In structural design and construction, the overall stability and reliability of the structure can be improved by defining the high sensitivity parameters of key parts and selecting appropriate materials to improve the parameter values.
3. For a homogeneous dam with a single material, the parameter sensitivity order of vertical displacement is *φ*_0_ > *K* > *R*_*f*_ > *K*_*b*_ > *n* >△*φ* > *K*_*ur*_ > *m*, and the parameter sensitivity order of horizontal displacement is *φ*_0_ > *K* > *K*_*b*_ > *n* > *R*_*f*_ > △*φ* > *K*_*ur*_ > *m*, which are basically the same as those obtained by orthogonal analysis and the Morris method. For dams with different material zones, the parameter sensitivity ranking of each material has some changes compared with that of dams with a single material; for example, the sensitivity ranking of the same kind of parameters of different material zones is different, and the sensitivity ranking of the parameters of each zone is not in complete accord. The difference in parameter level and material zoning is the reason for the difference in parameter sensitivity analysis results.

It is worth noting that the parameter sensitivity conclusions obtained in this paper are only applicable to this engineering case. For most CFRDs, displacements are more sensitive to *φ*_0_ and *K* and less sensitive to △*φ* and *m* [32–35]. However, due to the difference between the material zoning form of dam and mechanical parameter level of the dam material, as well as the selection of the number and position of characteristic nodes in the analysis, there must be certain differences in the parameter sensitivity ranking. Therefore, using the sPCE model for specific analysis is the most effective for different projects.

In addition, there are still some shortcomings in this study that need further exploration and analysis: (1) The minimum fitting sample size used in this study is 100 groups, which has high fitting accuracy and meets the analysis requirements. Nevertheless, when conducting complex analysis (such as finite element analysis of large 3D models or refined models under complex operating conditions), the calculation and collection of fitting samples will still consume too much effort. Therefore, determining the minimum fitting sample size that meets the accuracy requirements for different numbers of parameters is an important factor in improving the sensitivity analysis efficiency of the sPCE model. (2) In this case study, parameter sensitivity analysis is only conducted on the vertical and horizontal displacements in the typical cross section of the dam, excluding other cross section sections of the dam and transverse river displacements. And, the influence of dam foundation terrain on displacements and parameter sensitivity ranking are also ignored. This part needs to be considered in the design and construction of important engineering projects and important parts of the dam body.

## Supporting information

S1 Table Parameter sensitivity index of vertical displacement of each node in the typical cross section. (XLS)

S2 Table Parameter sensitivity index of horizontal displacement of each node in the typical cross section. (XLS)
